# Chicago Medical Society—Stated Meeting November 7th

**Published:** 1888-02

**Authors:** 


### CHICAGO MEDICAL SOCIETY.

### STATED MEETING, NOVEMBER 7, 1887.

The President, W. T. Belfield, M. D., in the Chair.

Professor J. A. ROBISON read a brief paper on the Treatment of Pertussis.

He said that since the discovery by Poulet in 1867 of a parasite which is probably the cause of whooping-cough, the disease has been treated by the local application of such germicidal agents as carbolic acid, eucalyptus, boracic acid, sulphur, illuminating gas, and resorcin. These had been applied by inhalations, insufflations, and sprays; but in many cases it is difficult to get children to allow the adminstration of the drugs; but he had used with success a solution of cocaine and resorcin in Semple's Atomizing Inhaler, the vapor being so fine that the drug is inhaled into the bronchial tubes without producing any laryngeal spasm. This method of treatment had been successful, not only in relieving the cough but in cutting short the course of the disease.

Professor F. E. WAXHAM said that he did not believe that we have a specific for the cure of this disease. He has tried sulphur fumigation thoroughly and considers it useless. He thinks that there are two indications to be met in the treatment of the disease — first, to destroy the bacilli as far as possible by means of germicides, as has been advised in the paper read; and second, to diminish the reflex excitability of the nervous system by the use of sedatives and tonics.

Professor G. C. PAOLI advocated the use of ergot in pertussis. In 15 cases it cut short the duration of the disease.

Professor JAMES H. ETHERIDGE read a paper on Vaginal Hysterectomy, with a report of three cases, which was given in the January number of the *Journal and Examiner*.

Professor C. T. PARKES, in discussing said that he was pleased with the paper, in that it introduces a method of controlling hæmorrhage that had been satisfactory to him a good many times. For this method we are indebted to Péan of Paris, who depends upon it on all occasions and in all operations. It is not an unusual thing to find a wound that he has made containing a dozen of these forceps. He never uses a drainage-tube. Professor Parkes says that he himself uses them in all operations where it is difficult to apply a ligature, and especially in removal of carcinoma of the rectum, on several occasions leaving a half dozen forceps, removing them in twenty-four hours, without subsequent trouble. He does not see the necessity of turning the uterus over, thus endangering the entrance of diseased tissue into the peritoneal cavity. The posterior wall is uncovered, the broad ligaments are exposed, and what necessity is there of turning the uterus over? These orceps will reach the top of it, and will include the broad ligaments on either side, and can be applied just as well with the uterus in position as if it were turned over.

Dr. D. T. NELSON desired to quote a word from Martin, of Berlin, spoken before the International Congress, where this subject was discussed quite fully. He advocates the operation strongly, and made the important point that the operation is not justifiable when the uterus is not movable. When the tissues above and about the uterus have become involved by the diseased condition, then the operation should not be done.

Professor C. T. PARKES reported a case of Nephrectomy. He said a patient had come under his care who, it was said, had been suffering for two years with cystitis, the diagnosis being based upon pus in the urine.

Upon examination, a tumor could be easily palpated in the right side of the body beneath the ribs, large enough to extend down to the superior spinous process of the ilium, and reaching up to the hypogastric region below the liver. The tumor appeared to be so elastic as to present some of the characteristics of a sac containing fluid. With an aspirator needle pus was detected. As the case presented none of the usual symptoms of a peri-nephritic abscess, it was diagnosticated a case of suppurative disease of the kidney, communicating with the bladder through the ureter. There was no apparent disease of the bladder itself, other than that which would be present as a consequence of the foreign substance in it.

In the operation an incision was made over the tumor outside of the erector spinæ muscle, and the tumor was exposed; then the pockets of pus in the organ were located by the hypodermic syringe. Three pockets of considerable size were opened, and drainage-tubes introduced. About a pint of pus was let out. It was decided that the three pockets did not communicate.

One of them opened freely into the pelvis of the kidney, and water injected into it went into the bladder, showing that there was a direct communication from this pus cavity with the bladder. The drainage-tubes were left in and the patient improved promptly, losing the fever and symptoms of pus accumulation and retention. For two weeks the improvement continued; then it was noticed that she began to fail rather rapidly and to show signs of fever again; there were signs of septic accumulation, and the tumor began to increase in size. As she was failing and the diagnosis was as complete as it was possible to make it, it was decided to perform nephrectomy.

There are some points of importance in the case: The drainage-tube that went into the pelvis of the kidney gave free exit to such a quantity of urine that it seemed almost the entire amount secreted by that kidney. There was also a free flow of urine from the bladder, and these facts led to the conclusion that the left kidney was not diseased and that the right could be removed.

Two or three hours before the operation was performed the patient was given gr. v. to x of quinine and gr. $\frac{1}{4}$ of morphine, to prevent shock, and the operation for the removal of the kidney was performed. The whole proceeding from begining to end occupied an hour, and she went to bed without any manifestation of shock, and with a pulse of 112. She had no rise of temperature until the second day, and then it rose to 100 F.; subsequent to that it fell to normal and did not rise above normal until the twelfth day, when other symptoms appeared. During all this time the wound was absolutely aseptic. It healed promptly by first intention, so that on the seventh day all stitches were removed.

The point of special surgical interest in the case was the manner of making the incisions. The commencement of the incision was two inches above the anterior superior spine of the ilium. It was carried in a curved direction downwards and backwards to the tip of the last rib. It was carried through all the tissues down to the fascia transversalis, everything having been carried forward out of the way, and with the finger the dissection was made well behind the tumor; all the parts were separated, then a straight incision made through all of them, straight back from the first incision and half way between the crest of the ilium and the last rib. The introduction of a ligature at the point of the posterior flaps and pulling them aside made an opening which admitted both hands.

By exposing the kidney completely, he could without difficulty reach the tissues which he wished to have under control.

As soon as the incision was made the tumor presented itself, and a finger could be carried around it in all directions so that the kidney with its blood-vessels, ureter and all were well exposed to view. Sixteen days after the operation the patient exhibited symptoms of cerebral trouble, with suppression of urine, and died with symptoms of uræmia.

At the time of the making of the report of the case no satisfactory miscroscopic examination of the tumor had been made, nor had the condition of the other kidney been demonstrated by the microscope. The post-mortem examination showed that it was congested and swollen, and ruptured capillary vessels in many places. Such examinations as had been made of several sections of it had not shown the presence of bacillus tuberculosis.

Dr. D. T. Nelson said that he was very much pleased at the doctor's instructions in regard to preparing the patient for these serious operations, and believed we have considerable to learn in that direction yet. He also suggested that the alimentary canal be prepared previous to such operations, and perhaps rendering it antiseptic or aseptic by emptying it and subsequently giving antiseptic drugs, preferably naphthalin in 5-grain doses, three times a day. He asked Professor Parkes if, in making the incisions, he is careful to preserve the capsule of the kidney and use that in facilitating drainage.

The president remarked that the fatal issue of this case through uræmia emphasizes the necessity for ascertaining the integrity of the opposite kidney before removing the one under suspicion; and mentioned the methods of obtaining urine of each kidney by cauterization of the ureter.

He said, it is questionable whether, for nephrectomy, or, indeed, all operations upon the kidney, chloroform is not preferable as an anæsthetic to ether, because of the decided renal irritation caused by the latter.

Professor PARKES, in closing the discussion, said that he considered the method of making the incisions superior to the old because of the increased freedom gained in getting at bleeding points caused by the enucleation.

In reference to Dr. Nelson's question as to whether he had cut down on the capsule, he said: "I am not positive whether I did or not. I pursued the course which I follow in removing all tumors, to cut down on the tumor and to get as close to it and as far away from the dangerous points as possible. I kept close to the surface of the kidney and away from the peritoneum.

"I do not know what would have been gained by Dr. Belfield's suggestion. This patient was secreting urine from the other kidney into the bladder; it was emptied out through the ureter and could be collected and examined just as well as if the ureter had been catheterized. In the first 24 hours the patient passed 7 ounces of urine from the bladder, in the next 24 hours 10 ounces, and so on up to the time of the occurrence of the unfortunate symptoms to which I have referred. The largest amount passed was 17.3 ounces in 24 hours. Up to that time there was nothing in the appearance of the patient that would make one think she was not going to recover. All at once the change and the end came."

